# A Dynamic Kalman Filtering Method for Multi-Object Fruit Tracking and Counting in Complex Orchards

**DOI:** 10.3390/s25134138

**Published:** 2025-07-02

**Authors:** Yaning Zhai, Ling Zhang, Xin Hu, Fanghu Yang, Yang Huang

**Affiliations:** 1 College of Information Engineering, Henan University of Science and Technology, Luoyang 471023, China; 2 Guangxi Technological College of Machinery and Electricity, Nanning 530007, China; 22012201007@mails.guet.edu.cn (Y.Z.); 22012302137@mails.guet.edu.cn (L.Z.); 3 School of Mechanical and Electrical Engineering, Guilin University of Electronic Technology, Guilin 541004, China; hx1263298586@mails.guet.edu.cn (X.H.); yangfanghu@mails.guet.edu.cn (F.Y.)

**Keywords:** target detection, multi-object tracking, Kalman filtering, fruit counting, intelligent orchard

## Abstract

With the rapid development of agricultural intelligence in recent years, automatic fruit detection and counting technologies have become increasingly significant for optimizing orchard management and advancing precision agriculture. However, existing deep learning-based models are primarily designed to process static and single-frame images, thereby failing to meet the large-scale detection and counting demands in the dynamically changing scenes of modern orchards. To address these challenges, this paper proposes a multi-object fruit tracking and counting method, which integrates an improved YOLO-based object detection algorithm with a dynamically optimized Kalman filter. By optimizing the network structure, the improved YOLO detection model provides high-quality detection results for subsequent tracking tasks. Then a modified Kalman filter with a variable forgetting factor is integrated to dynamically adjust the weighting of historical data, enabling the model to adapt to changes in observation and motion noise. Moreover, fruit targets are associated using a combined strategy based on Intersection over Union (IoU) and Re-Identification (Re-ID) features, improving the accuracy and stability of object matching. Consequently, the continuous tracking and precise counting of fruits in video sequences are achieved. Experimental results with image frames of fruits in video sequence are demonstrated, showing that the proposed method performs robust and continuous tracking (*MOTA* of 95.0% and *HOTA* of 82.4%). For fruit counting, the method attains a high coefficient-of-determination of 0.85 and a low root-mean-square error (RMSE) of 1.57, exhibiting high accuracy and stability of fruit detection, tracking and counting in video sequences under complex orchard environments.

## 1. Introduction

Intelligent agriculture is increasingly recognized as a vital approach for addressing sustainable food production challenges and enhancing orchard management efficiency [1]. Real-time tracking and counting of fruits have received significant attention [2,3,4], as accurate yield estimation is crucial for optimizing harvesting strategies and supply chain management. In this context, computer vision offers a promising non-invasive and highly accurate solution for fruit detection and quantification. Recent advances in deep learning and multi-sensor fusion [5,6] have accelerated the development of intelligent systems that operate reliably in dynamic orchard environments with varying light conditions, occlusion, and complex backgrounds. These computer vision-based fruit monitoring systems not only enable real-time yield prediction but also serve as critical components in achieving fully automated, data-driven intelligent orchard management [7].

Initially, fruit detection and counting relied on inefficient, costly manual methods. As machine vision technology advanced, traditional image processing techniques—including color segmentation [8] and edge detection [9]—found applications in orchard management. While these methods proved effective for uniformly colored fruits, they struggled with complex backgrounds and occlusions. Machine learning then introduced classifier models like Support Vector Machines (SVM) [10], Random Forests (RF) [11], and K-Nearest Neighbors (KNN) [12] for fruit detection. Though these models could accurately identify fruits using extracted image features, they were limited by their reliance on manual feature extraction and poor adaptability to real orchard environments.

Recent advances in deep learning-based Convolutional Neural Networks (CNNs) have enhanced environmental adaptability, detection accuracy, and computational efficiency. These networks excel at extracting high-dimensional image features through supervised learning while maintaining robustness against environmental interference. The YOLO (You Only Look Once) series has achieved remarkable detection accuracy and speed through continuous optimization, evolving from YOLOv5 to the latest YOLOv12 [13,14]. Specifically, YOLOv8 demonstrates exceptional capability in detecting small objects and handling complex scenes, making it particularly effective for real-time fruit detection where balancing accuracy and computational efficiency is critical [15]. Despite these advancements, existing YOLO-based methods predominantly focus on static detection tasks, often overlooking the temporal consistency required for tracking in dynamic agricultural environments. This limitation underscores the need for integrating robust tracking mechanisms to bridge the gap between detection and dynamic target monitoring. To address this challenge, Multi-Object Tracking (MOT) [16] technologies have been widely adopted in automated orchard management, with Tracking-by-Detection (TBD) [17] emerging as the dominant paradigm. Common TBD algorithms include Simple Online and Realtime Tracking (SORT) for basic tracking capabilities, DeepSORT with enhanced robustness through deep learning features, ByteTrack with improved occlusion handling, and BotSORT combining advanced techniques for better performance in changing scenarios [18].

Although existing algorithmic frameworks for MOT have achieved significant results, their performance is inherently dependent on the effectiveness of the underlying motion prediction models. Notably, these advanced trackers are all based on the Bayesian filtering theoretical system, among which Kalman filtering (KF), as a classical method, is designed for linear Gaussian systems, which realizes efficient targets through recursive estimation of the state and observation equations tracking [19]. Compared to its variants Extended Kalman filtering (EKF) and Untraceable Kalman filtering (UKF), in nonlinear scenarios, KF, with its strict linearity assumption and O(n^3^) time complexity (n is the state dimension), has a significant advantage in computational efficiency [20]. This property makes it particularly suitable for resource-constrained environments such as orchards, where the fruit motion usually exhibits an approximately uniform linear trajectory. In addition, YOLOv8n can maintain high accuracy detection in complex occlusion scenarios through the improvement of the lightweight architecture and attention mechanism [15], which provides new possibilities for building a collaborative “detection-tracking” framework: by combining the detection output of YOLOv8n with the KF-based tracking module, the timing consistency can be effectively improved, and the real-time requirement can be met.

In the advancement toward intelligent orchard management, deep learning-based fruit counting methods have been extensively explored using both single-image and video sequence paradigms. Among these, a video sequence-based algorithm integrating OrangeYOLO with OrangeSORT [21] has demonstrated superior performance over conventional tracking algorithms such as SORT and DeepSORT. Additionally, an enhanced Hungarian algorithm combined with YOLOv4-tiny has achieved a tracking accuracy of 69.14% in apple detection and counting tasks [22]. Recent studies have also successfully employed YOLOv5 and DeepSORT in conjunction with unmanned aerial vehicles (UAVs) for tomato flower and fruit monitoring [23]. Building upon these foundations, recent advances have utilized improved YOLO architectures combined with adaptive Kalman filtering for bagged grape counting. These methods typically integrate feature enhancement modules (e.g., EFEM, SEAM) to improve occlusion resistance in detection, while dynamic noise estimation algorithms enhance tracking robustness against camera shake and rapid motion. Comparative studies indicate that such hybrid systems achieve higher tracking accuracy and counting precision compared to traditional YOLO baselines, highlighting their potential for complex orchard environments [24]. While these approaches have shown considerable effectiveness across various horticultural applications, several challenges persist in complex orchard environments: (1) Densely clustered fruits with high visual similarity can increase identity mismatches, degrading tracking accuracy; (2) Extended occlusion of target fruits may result in track fragmentation and accumulated errors, diminishing long-term tracking performance; (3) Motion blur from camera movement or mobile platforms can significantly interrupt trajectories, affecting the continuity and reliability of fruit counting.

To address these challenges, this study proposes a novel approach for fruit tracking and counting in complex orchard environments. The methodology integrates an improved YOLO-based detection algorithm with a BotSORT-based tracking system [25] and a dynamic optimized Kalman filter by incorporating a dynamic forgetting factor that adjusts the weighting of historical data over time. These adjustments account for variations in observation and motion noise, improving estimation accuracy and enhancing adaptability to changing dynamics. The optimized Kalman filtering technique minimizes tracking disruptions, ensuring continuous fruit monitoring across video sequences. Additionally, the system combines Intersection-over-Union (IoU) and Re-Identification (Re-ID) techniques for target association, enabling more stable and accurate fruit tracking through enhanced trajectory updates. The remainder of the paper offers the following:

- The developed fruit tracking and detection model, including improvements to the network architecture through enhanced feature extraction modules, optimized detection heads, and integrated attention mechanisms are demonstrated.
- The construction of the training dataset using appledatasets and Synthetic-apples for model training and testing is explained.
- The results that evaluate the performance of the fruit detection module (using an improved YOLOv8 model) and the fruit tracking module (with enhanced Kalman filtering) are presented.

## 2. Dynamic Kalman Filtering Method for Multi-Object Fruit Tracking and Counting

Deep learning-based fruit object detection holds significant importance in monitoring fruit growth and estimating yields. However, in complex orchard environments, fruit object detection using deep learning techniques still faces challenges such as small and densely packed fruit targets with occlusion and difficulties in accurately identifying multiple fruits. As one of the latest versions in the YOLO series, YOLOv8 has demonstrated excellent performance in detecting small targets and handling complex scenarios. Particularly for real-time fruit detection tasks, YOLOv8 can effectively balance detection speed and precision, providing stronger support for fruit detection and monitoring [26]. Consequently, this study selects YOLOv8n as the baseline network model for apple detection in complex orchard settings.

To address the challenges of detecting small and occluded fruit targets in complex orchard environments, this paper proposes a refined YOLOv8n architecture with three key improvements: EfficientNet-B0 [27] is adopted as the backbone network to enhance feature extraction capabilities, Multi-Scale Dilated Attention (MSDA) [28] is integrated to mitigate occlusion-related detection errors, and Four Adaptively Spatial Feature Fusion (FASFF) [29] is designed as the detection head to optimize multi-scale target localization. These architectural enhancements collectively improve detection accuracy, particularly for small and heavily occluded targets. The dynamic Kalman filter is optimized in both motion prediction and appearance association by introducing a variable forgetting factor, which dynamically adjusts the process noise covariance matrix. This approach effectively mitigates trajectory prediction biases caused by fixed parameters in traditional Kalman filters and enables automatic adaptation to abrupt or irregular fruit movements, thereby improving the accuracy and robustness of motion prediction. In conjunction with detection box updates, a camera motion compensation technique is introduced to attenuate camera motion interference and ensure tracking continuity, thereby improving prediction accuracy. Furthermore, to enhance the precision of multi-object tracking, this paper implements an Intersection-over-Union and Re-Identification fusion strategy that effectively reduces ID confusion in high-density fruit scenarios by combining spatial and appearance cues—where IoU provides reliable geometric constraints for closely spaced fruits while Re-Identification distinguishes visually similar targets through deep feature matching. The synthesis of these enhancements leads to a robust and high-performance system capable of accurately tracking and counting fruits within complex orchard environments.

**Figure 1:**
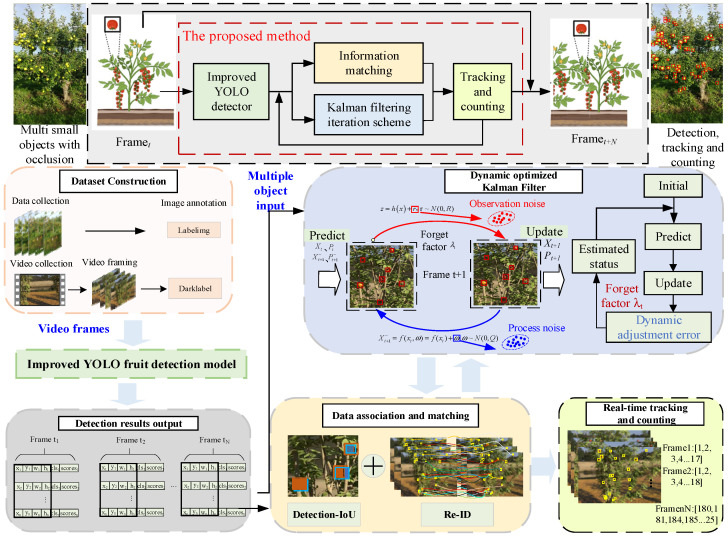
Dynamic Kalman filtering method for apple detection and counting.

As shown in Figure 1, the methodology proposed in this paper mainly includes four processes: (1) Detection of fruits in the video sequences by the object detection model with the improved YOLOv8n, where the red boxes indicate the detection results; (2) Feature extraction and state prediction of appearance, position, and motion characteristics of the fruit using the dynamic Kalman filter; (3) Data association and matching based on target detection results and state prediction; (4) Fruit counting by combining continuous tracking results to count the number of fruits, providing a basis for yield estimation.

### 2.1. Dataset Construction

**Figure 2:**
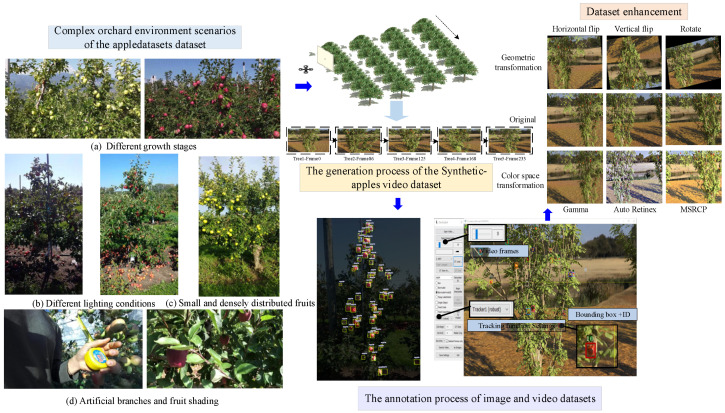
Dataset construction.

The effectiveness of the proposed method was experimentally validated with the open source appledatasets, comprising 7311 images, as illustrated in Figure 2. These datasets presented challenging factors such as small targets, occlusions, dense targets, and a complex environment in the orchard. The LabelImg annotation tool was employed for labeling.

The training, validation, and test sets were randomly divided in a 7:2:1 ratio, which facilitates model generalization and provides more compelling evidence for the improved multi-target tracking method. Regarding the video dataset, the Synthetic-apples dataset is selected with a duration of 24 s and a frame rate of 10 frames per second. The video contained 250 frames and five trees. As shown in Figure 2, the Darklabel annotation tool was used to manually annotate the bounding boxes and IDs of the 250 frames. The data was saved in the MOT data format and used as ground-truth reference data to verify the tracking and counting performance.

To enhance the diversity of the data and simulate the different lighting conditions and viewpoint changes that may occur in the orchard environment, data was further performed including flipping, rotation, scaling, noise addition, and color contrast enhancement. This approach helped improve the robustness of the fruit target detection model when facing diverse lighting, posture, and background conditions, thereby enhancing its adaptability to different practical application scenarios.

### 2.2. Fruit Detector by the Improved YOLO Model

In an agricultural environment, fruit detection frequently involves targets characterized by multi-scale variations, dense arrangements, and mutual occlusion. While YOLOv8 is a prevalent real-time object detection framework, it typically relies on the Darknet53 architecture for the backbone network, which may limit its capacity to fully optimize network depth, width, and resolution. This limitation can limit the effective extraction of multi-scale features while maintaining computational efficiency, potentially causing an imbalance between detection accuracy and computational demands.

**Figure 3:**
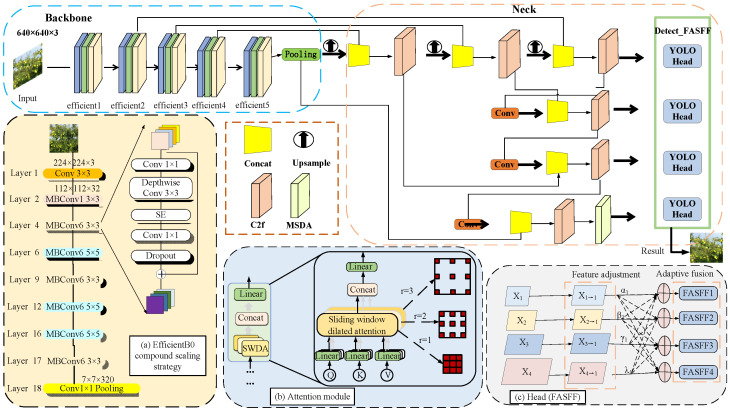
YOLO fruit detection model. (**a**) EfficientB0 network architecture, (**b**) MSDA attention module, (**c**) FASFF module.

To address these limitations, this study refines the network architecture by modifying the feature extraction module, optimizing the detection head and integrating an attention mechanism, as illustrated in Figure 3. Specifically, the EfficientNetB0 module is implemented as the backbone network to enhance feature extraction through a novel unified scaling method. An ASFFHead four-head structure is designed to improve cross-scale feature fusion, particularly for small target detection. Additionally, the multi-scale dilated attention (MSDA) mechanism is incorporated to enhance detection accuracy and processing efficiency.

In the modified backbone architecture, EfficientNetB0 is implemented to replace the Darknet53 network in YOLO. EfficientNetB0, recognized for its efficiency in convolutional neural network design, balances network complexity during its architectural design. By employing a compound scaling strategy, EfficientNetB0 uniformly scales the network’s depth, width, and resolution. This design enhances adaptability to varying data conditions across diverse scenarios and demonstrates robust feature extraction capabilities. Moreover, a unified scaling method MBConv module is utilized, as shown in Figure 3a. A 1 × 1 standard convolution is integrated for upscaling, alongside a 3 × 3 depthwise convolution, a Squeeze-and-Excitation module for implementing attention weighting, a 1 × 1 standard convolution for downscaling, and a Dropout layer. This design effectively minimizes the model’s parameter count and computational demands, leading to faster processing and greater computational efficiency, while maintaining a lightweight model profile suitable for real-time fruit detection applications.

To address the trade-off between computational demands and field-of-view limitations in global attention mechanisms, this study introduces the MSDA module. Inspired by the Vision Transformer (ViT) architecture, the MSDA (multi-scale dilated attention) module is designed to emulate localized and sparse interactions across varying scales. By employing a parallel arrangement of dilated convolution layers with differing dilation rates, the module enhances the capacity to capture features from targets at multiple scales and enriches the information within the feature maps, as depicted in Figure 3b. This approach facilitates improved identification of occluded apples and enables effective differentiation of individual fruits within densely packed areas, thereby optimizing the accuracy of apple counting and tracking throughout the orchard environment.

To solve the variations in fruit characteristics across different scales, this paper incorporates the adaptively spatial feature fusion (ASFF) module into the YOLOv8n detection head. This incorporation is then further innovated by introducing a fourth output layer. This is further enhanced by introducing a fourth output layer, transforming the conventional three-head detection structure into a four-head configuration, designated as the FASFF (Four Adaptively Spatial Feature Fusion) module as shown in Figure 3c. This modification serves to diminish feature conflicts, compensate for feature attenuation resulting from cross-scale fusion and enhance the secondary extraction capabilities of small target detection layers. With the introduction of the FASFF module, the enhanced feature extraction process effectively addresses the problems associated with detecting fruits of varying sizes and densities within the complex orchard environment.

### 2.3. Fruit Tracking and Counting Modules

#### 2.3.1. Dynamic Kalman Filtering Method

In this study, we choose to adopt the standard Kalman Filter (KF) for motion prediction rather than using more complex nonlinear Bayesian filters such as the Extended Kalman Filter (EKF) or the Unscented Kalman Filter (UKF). This choice is based on three main considerations. First, the motion of fruits between consecutive video frames in orchard environments can typically be well-approximated by a constant-velocity model over short time intervals. Therefore, the linear assumptions underlying the KF are sufficient to model this motion accurately. Second, the KF offers high computational efficiency, with a time complexity of approximately ***O***(*n*^3^), which is well-suited to the real-time processing requirements of fruit tracking systems. In contrast, EKF and UKF introduce significantly greater computational overhead—typically two to three times higher—due to the need for Jacobian matrix calculations or sigma point propagation, which can lead to undesirable latency. Third, the KF is fully compatible with widely used multi-object tracking (MOT) frameworks such as SORT, DeepSORT, ByteTrack, and BotSORT, all of which rely on linear motion models. In contrast, integrating nonlinear Bayesian filters into these frameworks would require extensive architectural modifications and could compromise system stability or performance. For these reasons, we focus on enhancing the robustness of the KF, specifically by introducing a dynamic forget factor, rather than replacing it with nonlinear alternatives.

The dynamic Kalman filtering method with a variable forgetting factor proposed in this paper includes three main stages: initialization, prediction, and update.

In the initialization stage, the above-mentioned YOLO object detection model is used to detect fruits in the *t*th frame of image sequences. The fruit’s position information, including coordinates, category labels, and confidence scores is obtained. The initial state vector **X_t_** and initial covariance matrix **C_t_** defined in Equations (1) and (2) can be calculated.

(1) $$X_{t}={\left[p_{t},v_{t}\right]}^{T}$$

(2) $$C_{t}=diag\left(\begin{array}{c} \sigma_{p}^{2}I_{4\times 4},\sigma_{v}^{2}I_{4\times 4} \end{array}\right)$$

where the state vector **X_t_** consists of the position *p_t_* and velocity state *v_t_* variables of the detection box defined in (3). (*x_t_*, *y_t_*) represent the center position coordinates of the detection box at time-frame *t.* And (*w_t_*, *h_t_*) are the width and height of the detection box. (*v_xt_*, *v_yt_*, *v_wt_*, *v_ht_*) are the corresponding velocities, respectively.

(3) $$\left\{\begin{array}{l} p_{t}={\left[\begin{array}{c} x_{t},y_{t},w_{t},h_{t} \end{array}\right]}^{T}\\ v_{t}={\left[\begin{array}{c} v_{xt},v_{yt},v_{wt},v_{ht} \end{array}\right]}^{T} \end{array}\right.$$

The measurement uncertainties in (2) are represented by ($\sigma_{p}^{2}$, $\sigma_{v}^{2}$), calculated by the initial measurement noise weight *w* in (4) and (5). (*w_p_*, *w_v_*) denote the noise weight for position and velocity, respectively. In general, the noise weight for velocity is higher than that for position, as velocity is derived through differentiation. So the different values of 2 and 10 are set in (5) for the position and velocity, respectively. These specific values (2 for position and 10 for velocity) are empirically determined based on both the motion characteristics of fruits in orchard environments and the Kalman filter design in the BoT-SORT algorithm [25], where they were selected through extensive experimental tuning on public MOT datasets (e.g., MOT17). The covariance matrix ***C_t_*** is initialized as a diagonal matrix, where the diagonal elements are the squared standard deviations of the respective variables. The off-diagonal elements are set to 0, indicating that the variables are initially uncorrelated. *I*_4×4_ represents a 4 × 4 identity matrix.

(4) $$\left\{\begin{array}{l} \sigma_{p}^{2}=\sigma_{px}^{2}+\sigma_{py}^{2}\\ \sigma_{v}^{2}=\sigma_{vx}^{2}+\sigma_{vy}^{2} \end{array}\right.$$

(5) $$\left[\begin{array}{c} \sigma_{px}\\ \sigma_{py}\\ \sigma_{vx}\\ \sigma_{vy} \end{array}\right]=\left[\begin{array}{cccc} 2 & 0 & 0 & 0\\ 0 & 2 & 0 & 0\\ 0 & 0 & 10 & 0\\ 0 & 0 & 0 & 10 \end{array}\right]\left[\begin{array}{c} w_{p}w_{t}\\ w_{p}h_{t}\\ w_{v}w_{t}\\ w_{v}h_{t} \end{array}\right]$$

In the prediction stage, the state vector **X_t_** (8 × 1) and covariance matrix **C_t_** (8 × 8) of the target at the previous time are used to estimate the predicted position and velocity of the target in the current frame ($X_{t+1}^{-}$, $C_{t+1}^{-}$) through the state transition model, as shown in Equations (6) and (7).

(6) $$X_{t+1}^{-}=FX_{t}+W,W∼N(0,\;Q)$$

(7) $$C_{t+1}^{-}=FP_{t}F^{T}+Q$$

where *W* denotes the process noise vector to account for the random disturbances and unpredictable factors in the motion of the target. **Q** is the covariance matrix of process noise used to quantify the error introduced by motion uncertainties during the prediction step. The definition of **Q** relies on the standard deviations of the target’s position and velocity states, which directly impact the accuracy of the predicted state. $X_{t+1}^{-}$ and $C_{t+1}^{-}$ are the predicted state vector and covariance matrix, respectively. These predicted values serve as a foundation for the subsequent Kalman filtering update, ensuring that the tracking model closely follows the dynamic changes in the detected target.

In (6), *F* is a 8 × 8 state transition matrix for state update in the prediction stage. *F* is obtained by linearization in (8)–(10) under the assumption of uniform motion, which effectively captures the target’s motion dynamics.

(8) $$X_{t+1}^{-}=f(X_{t},W)=f(X_{t})+W$$

(9) $$F=\left[\begin{array}{cc} I_{4\times 4} & \Delta t\cdot I_{4\times 4}\\ 0 & I_{4\times 4} \end{array}\right]$$

where *f*(**X_t_**, *W*) represents the state transition function, which captures the deterministic component of the motion dynamics of target. The term *f*(**X_t_**) denotes the deterministic part of the state transition. Furthermore, the process noise *W* is modeled as a zero-mean Gaussian distribution with a standard deviation of **Q**, accounting for the random disturbances and unpredictable factors that influence the motion. ∆*t* in (9) is the time interval. Therefore, Equation (6) for predicting the state vector $X_{t+1}^{-}$ can be expanded into the linear form:

(10) $$\left\{\begin{array}{l} x_{t+1}=x_{t}+v_{x_{t}}\Delta t,\;y_{t+1}=y_{t}+v_{yt}\Delta t\\ w_{t+1}=w_{t}+v_{wt}\Delta t,\;h_{t+1}=h_{t}+v_{ht}\Delta t\\ v_{x_{t+1}}=v_{x_{t}},\;v_{y_{t+1}}=v_{y_{t}}\\ v_{w_{t+1}}=v_{w_{t}},\;v_{h_{t+1}}=v_{h_{t}} \end{array}\right.$$

**Figure 4:**
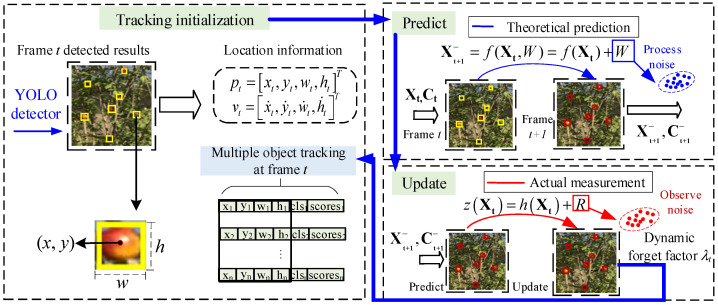
Illustration of the dynamic Kalman filtering method with a variable forget factor.

In the update stage, the current states are combined with the detected observation information to correct the predicted state vector $X_{t+1}^{-}$ and covariance matrix $C_{t+1}^{-}$ to obtain the updated state vector **X_t+1_** and covariance matrix **C_t+1._** By computing the Kalman gain, the updated state vector **X_t+1_** and covariance matrix **C_t+1_** can more precisely track the actual position and motion state of the target. This correction process integrates the motion information with the prediction model and the measurement information from the observation model, consequently enhancing the accuracy and robustness of the tracking in (11)–(15). The whole stages of the proposed dynamic Kalman filtering method with a forget factor are illustrated in Figure 4, where the yellow and red boxes denoting the predicted and updated results respectively. 

With the calculated predicted state vector $X_{t+1}^{-}$ and covariance matrix $C_{t+1}^{-}$, the Kalman filter gain can be obtained using (11). Then the updated state vector **X_t+1_** can be determined in (15) with current observation value $z(X_{t+1}^{-})$ and predicted value $HX_{t+1}^{-}$ accounting for the process noise.

(11) $$K=\frac{C_{t+1}^{-}H^{T}}{HC_{t+1}^{-}H^{T}+S}$$

(12) $$S=diag\left(\begin{array}{c} \sigma_{p}^{2}I_{4\times 4} \end{array}\right)$$

(13) $$H=[I_{4\times 4},0]$$

(14) $$z(X_{t+1}^{-})=h\left(X_{t+1}^{-}\right)+R,R∼N(0,S)$$

(15) $$X_{t+1}=X_{t+1}^{-}+K[z(X_{t+1}^{-})-HX_{t+1}^{-}]$$

where *z*(**X_t_**) is the observation value. *R* is the observation noise that follows the zero-mean Gaussion distribution with the standard deviation of **S**. **S** is the observation noise covariance matrix, which describes the uncertainty of the observation. *h*(**X_t_**) is the observation function. *K* is the Kalman filter gain. **H** is the observation matrix while *I*_4×4_ represents a 4 × 4 identity matrix.

To enhance the adaptability of the dynamic Kalman filter method, this paper developed a variable forget factor *λ_t_*, which dynamically updates the covariance matrix as follows:

(16) $$C_{t+1}=(I-KH)\frac{C_{t+1}^{-}}{\lambda_{t}}+(1-\frac{1}{\lambda_{t}})C_{t}$$

where $\lambda_{t}\in [0,1]$ is the forget factor, and **C***_t_* is the covariance matrix in the previous frame. The dynamic adjustment allows the system to respond flexibly to changes in different noise levels during tracking.

The variable forget factor is dynamically adjusted based on the squared norm of the measuring residual *δ_t_* according to (17) and (18).

(17) $$\lambda_{t}=\lambda_{min}+(\lambda_{max}-\lambda_{min})\cdot exp(-\gamma \cdot ||\delta_{t}|{|}^{2})$$

(18) $$||\delta_{t}|{|}^{2}={\left[z(X_{t})-HX_{t}^{-}\right]}^{T}\left[z(X_{t})-HX_{t}^{-}\right]$$

where *γ* is the adjustment parameter that controls the rate of change in the forget factor in the range $0<\lambda_{min}\leq \lambda_{t}\leq \lambda_{max}\leq 1$.

**Figure 5:**
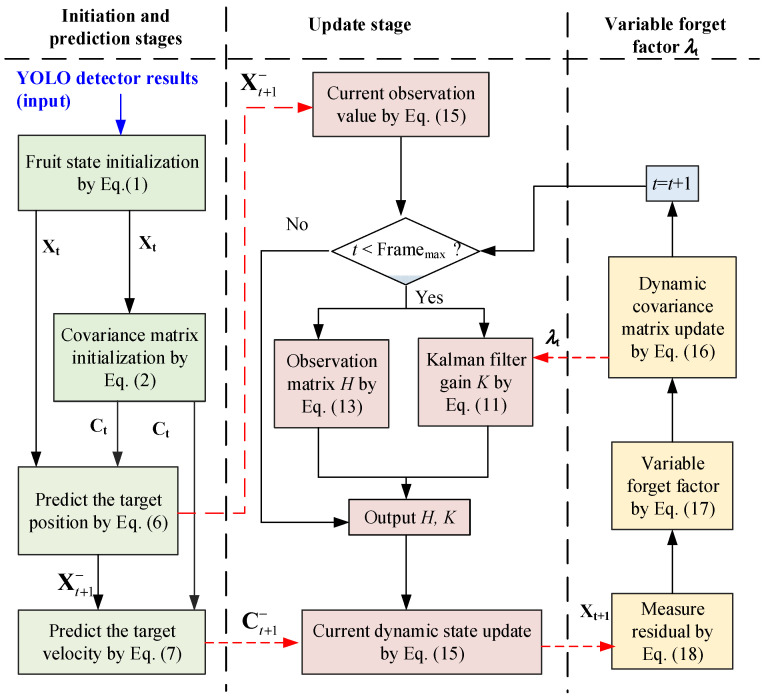
Flow-chart of the dynamic Kalman filtering method.

The proposed Kalman filtering method incorporating a variable forget factor allows the system to dynamically adjust the update strategy based on changing noise conditions, resulting in enhanced robustness and precision for target tracking in an orchard environment. By analyzing the measurement residual in real-time, the filter can reduce the dependence on historical data in high-noise environments, while fully leveraging valuable data in low-noise scenarios to further enhance state estimation accuracy. This dynamic adaptation process enables the system to adapt to diverse and challenging real-world conditions. This correction process, which combines the motion information of the prediction model and the measurement information of the observation model, is crucial for ensuring tracking accuracy and adaptability to the dynamic changes in the target. The flow-chart of the dynamic Kalman filtering method is illustrated in Figure 5, where the dotted lines the parameter transfer relationships between stages.

#### 2.3.2. Camera Motion Compensation Method

In an orchard fruit tracking environment, camera motion can introduce deviations in the predicted locations of the bounding boxes of the target by YOLO, consequently impacting the accuracy of tracking. To address this issue, the proposed method employs a camera motion compensation method with local multi-target detection to correct the bounding box positions by sparse optical flow.

Under the assumptions of constant brightness and small pixel displacement, the optical flow method estimates the motion of objects between two consecutive frames by analyzing the changes in image brightness patterns. With the detected positions of fruit targets in consecutive frames (*p_i_*, *q_i_*), the camera motion can be described by a comprehensive transformation matrix *A* that captures various affine transformations.

(19) $$q_{i}=A\cdot \left[\begin{array}{c} p_{i}\\ 1 \end{array}\right]=\left[\begin{array}{ccc} a_{11} & a_{12} & a_{13}\\ a_{21} & a_{22} & a_{23} \end{array}\right]\left[\begin{array}{c} x_{i}\\ y_{i}\\ 1 \end{array}\right]$$

where *p_i_* and *q_i_* are the feature-positions of multi-targets in the current and previous frame, respectively. *a*_11_–*a*_23_ are the affine transformation parameters accounting for the rotation, scaling, translation, and shear.

To enhance the accuracy and robustness of this motion estimation, the transformation matrix *A* is solved using the RANSAC (random sample consensus) iterative algorithm using the feature-positions of multiple detected fruit targets. This approach ensures a more reliable estimation of the movement of movement. According to calculated transformation matrix *A*, the predicted center position of each bounding box in the current frame can be compensated by multiplying it with the inverse of *A*. This compensation mitigates the impact of camera motion, enabling the accurate tracking of fruit targets despite the dynamic camera movement inherent in the orchard environment.

(20) $$\left[\begin{array}{c} x_{c}\\ y_{c} \end{array}\right]=A^{-1}\cdot \left[\begin{array}{c} x\\ y\\ 1 \end{array}\right]$$

(21) $$\left[\begin{array}{c} w_{c}\\ h_{c} \end{array}\right]=\left[\begin{array}{cc} s_{x} & 0\\ 0 & s_{y} \end{array}\right]\left[\begin{array}{c} w\\ h \end{array}\right]$$

where (*x*, *y*) are the center coordinates and (*w*, *h*) are the width and height of the bounding boxes calculated by the Kalman filter. (*x_c_*, *y_c_*, *w_c_*, *h_c_*) are the compensated state information. (*s_x_*, *s_y_*) are the scaling factors in matrix *A*.

#### 2.3.3. Data Association and Matching Method

To further enhance the accuracy of multi-target fruit tracking, an IoU-Re-ID fusion strategy is developed. First, the FastReID library is utilized to extract the Re-ID features of the targets. Then, an exponential moving average method is employed to smooth the features, with the average appearance feature state of the trajectory (tracklet) updated in real-time according to the equation:

(22) $${\bar{f}}_{t+1}=\alpha {\bar{f}}_{t}+(1-\alpha )f_{t+1}$$

where *α* (0 < *α* < 1) is the smoothing coefficient. ${\bar{f}}_{t+1}$ is the smoothed feature, and *f_t_*_+1_ is the current feature.

Moreover, the IoU and Re-ID features are fused to optimize the target matching accuracy by simultaneously considering the motion information and appearance information of the targets. Specifically, IoU is used to measure the degree of overlap of the targets in space, while the Re-ID feature is employed to assess the similarity of the target appearance. The IoU and Re-ID feature similarity are calculated separately to capture the motion and appearance information of the targets. Subsequently, the low-relevance IoU matching candidates are filtered out through Re-ID. The association matrix is then generated by fusing IoU and Re-ID features based on the minimum value rule:

(23) $$\left\{\begin{array}{c} d_{IoU}(A,B)=1-\frac{Area(A\cap B)}{Area(A\cup B)}\\ d_{ReID}(a,b)=1-\frac{a\cdot b}{\left||a||\;||b|\right|}\\ d_{fused}=min\left(d_{IoU},d_{ReID}\right) \end{array}\right.$$

**Figure 6:**
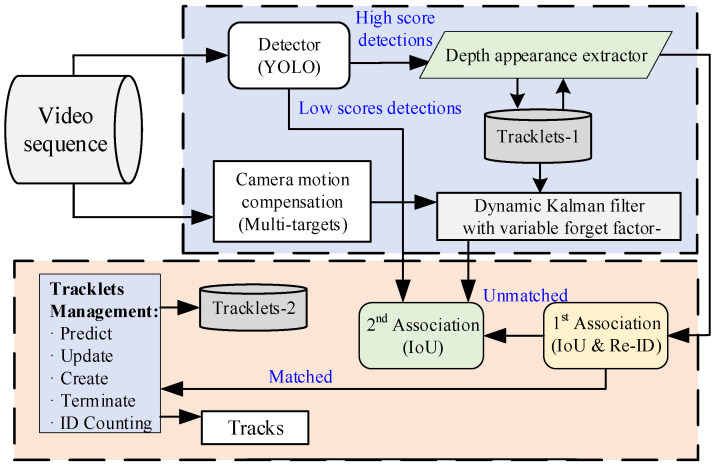
Flow-chart of the dynamic Kalman filtering tracker of fruit.

The proposed dynamic Kalman filtering tracker of fruit has implemented multiple optimizations in motion prediction and appearance association. By optimizing both spatial and appearance dimensions simultaneously, this approach can improve the overall performance of target tracking with enhanced *MOTA* (multiple object tracking accuracy) and *IDF1* (ID F1 score) indicators. First, the proposed method utilizes a Kalman filter with a variable forget factor to predict the motion trajectory of the fruit. Then novel detection boxes for the update are combined with the Kalman filter while implementing a camera motion compensation method to handle the interference of camera motion, ensuring the continuity of tracking and improving prediction accuracy. Finally, IoU and ReID are employed for association to match the target fruit and update the trajectory. The YOLO-based dynamic fruit detection and tracking method can achieve more efficient and accurate fruit tracking. The overall process is illustrated in Figure 6.

### 2.4. Experimental Environment and Evaluation Metrics

The hardware used in this experiment included a 128-core AMD EPYC 9754S processor (Advanced Micro Devices, Inc., Santa Clara, CA, USA) with 128 GB of RAM, and an NVIDIA GeForce RTX 4090 graphics card with 24 GB of video memory (NVIDIA Corporation, Santa Clara, CA, USA). The software environment was based on the PyTorch 1.12.1 deep learning framework, CUDA version 11.6 and Python 3.8.

To evaluate the performance of the developed fruit detector, the evaluation metrics of *mAP* (mean Average Precision), GFLOPs (Giga Floating-point Operations Per Second), FPS (Frames Per Second), and model size were used. *mAP* is a comprehensive metric that reflects the accuracy and recall of the model. GFLOPs is used to measure the computational complexity of the model, i.e., the number of floating-point operations required.

(24) $$mAP=\frac{1}{n}\sum_{i=1}^{n}\int_{0}^{1}P(R)dR$$

(25) $$FPS=n_{frame}/t_{elapse}$$

where in (24) *P* is precision and *R* is recall. *n* is the total detection number of frame. *FPS* in (25) is the number of frames divided by the elapsed time denoting the computational efficiency.

To analyze the performance of the fruit tracker, multiple indicators were used. Among them, the three most important indicators were *MOTA*, *IDF1* and *HOTA*. *MOTA* (Multiple Object Tracking Accuracy) considers false positives, false negatives, and identity switches to measure overall accuracy; *IDF1* (ID F1 score) was used to measure the accuracy of target ID recognition for each tracking box, taking into account both the accuracy and recall of the target ID; *HOTA* (Higher Order Tracking Accuracy) was an extension of *MOTA*, which combines various metrics to evaluate different types of errors.

(26) $$MOTA=1-\frac{\sum \left(n_{FN}+n_{FP}+n_{IDSW}\right)}{n_{GT}}$$

(27) $$IDF1=\frac{2n_{IDTP}}{2n_{IDTP}+n_{IDFP}+n_{IDFN}}$$

(28) $$HOTA=\sqrt{\frac{\sum A(c)}{\left|n_{TP}|+|n_{FN}|+|n_{FP}\right|}},c\in \{TP\}$$

where *n_FN_* is the number of false negatives (i.e., missed ground truth objects that were not detected). *n_FP_* is the number of false positives (i.e., falsely detected objects that do not match any ground truth). *n_GT_* is the total number of ground truth objects across all frames. *n_IDSW_* is the number of identity switches (i.e., times that a tracked object’s identity changes during tracking). *n_IDTP_* is the number of ID true positives (i.e., detection with correct ID). *n_IDFP_* is the number of ID false positives (i.e., detection assigned the wrong ID). *n_IDFN_* is the number of ID false negatives (i.e., ground truth instances that were not assigned any correct tracking ID). *A*(*c*) is the association accuracy for correct match *c*, where *c* belongs to the true positives. (|*n_TP_*|, |*n_FN_*|, |*n_FP_*|) is the total number of true positives, false negatives, and false positives, respectively.

To statistically evaluate the performance of the proposed method by comparing the results from test videos with manually counted ground truth, the coefficient of determination *R*^2^ and the root-mean-square error (RMSE) were used.

(29) $$R^{2}=1-\frac{\sum_{t=1}^{N}{\left(n_{t}-{\overset{⌢}{n}}_{t}\right)}^{2}}{\sum_{t=1}^{N}{\left(n_{t}-\bar{n}\right)}^{2}}$$

(30) $$RMSE=\sqrt{\frac{1}{N}\sum_{t=1}^{N}{\left(n_{t}-{\overset{⌢}{n}}_{t}\right)}^{2}}$$

where *n_t_* is the actual number of fruit in time-frame *t* and ${\overset{⌢}{n}}_{t}$ is the counted number of fruit by the proposed method at *t*. $\bar{n}$ is the mean of the actual number of fruit while *N* is the total number of fruit.

## 3. Results and Discussion

### 3.1. Comparisons of YOLO Detection Model

To evaluate the generalization capability of the improved YOLO model proposed in this paper, comparative experiments were conducted by training YOLOv5n, YOLOv7, and YOLOv8n separately on the appledatasets dataset. The experimental results were summarized in Table 1.

As indicated in Table 1, the YOLOv7 model had the lowest *mAP*@0.5 value and the highest GFLOPs, indicating poor detection accuracy combined with high computational complexity. The lack of model compactness in YOLOv7 makes it unsuitable for the experimental environment in fruit tracking and counting. In contrast, YOLOv5 exhibited the fastest detection speed and smaller GFLOPs value. However, its *mAP*@0.5 of 84.1% was still lower than that of the YOLOv8n, limiting its detection performance. The YOLOv10n model achieved a *mAP*@0.5 of 83.1% with a GFLOPs of 8.2, which did not demonstrate a clear advantage in either accuracy or efficiency. YOLOv11 and YOLOv12n achieved slightly better accuracy with *mAP*@0.5 values of 84.2% and 83.5%, respectively, while maintaining lower GFLOPs (6.3 and 6.0), indicating reduced computational complexity. Nevertheless, these models still fell short of YOLOv8n in terms of detection accuracy. Overall, the YOLOv8n model achieved a balanced performance with a *mAP*@0.5 of 85.8% and a GFLOPs of 8.1, offering a favorable trade-off between detection accuracy and computational cost. This makes it a more suitable baseline for our application scenario involving real-time fruit tracking and counting under complex orchard conditions.

To further validate the effectiveness of the improved modules proposed in this paper, ablation experiments were conducted on the appledatasets with a total of seven models. The experimental environment and parameters were all the same but with different modules between models in Table 1.

In Table 1, model 1 replaced the backbone network of YOLOv8n with the EfficientNetV1 structure. The compound scaling method was applied to uniformly scale the network depth, width, and resolution, which significantly reduces the number of model parameters and computational cost while maintaining model accuracy, resulting in a 1.3% improvement in average detection accuracy compared to the original YOLOv8n network. Model 2 introduced the MSDA module based on the baseline network YOLOv8n, leading to a 0.5% increase in average detection accuracy. Model 3 modified the detection head to ASFFHead and added a 4th input layer, resulting in a 2.9% improvement in average detection accuracy. Model 4 combined the MSDA module on the basis of model 1. With a negligible increase in GFLOPs, model 4 had a 1.4% increase in precision with model 1. Based on model 3, Model 5 and 6 incorporated the EfficientNetV1 and MSDA modules, respectively, resulting in an enhancement in the average fruit detection accuracy, with improvements of 2.4% and 2.5%, respectively. In comparison, the proposed method (Model 7) achieved the best overall detection performance, attaining a *mAP*@0.5 of 89.5% (a 3.7% improvement over the baseline model) and a *mAP*@0.5:0.95 of 47.5%, representing a 5.0% increase. Notably, this performance gain was achieved while maintaining acceptable computational complexity at 12.9 GFLOPs, demonstrating a balanced trade-off between accuracy and efficiency. These findings validated the effectiveness of the improvements made to the detection model. The improved YOLO method proposed in this paper exhibited good performance and can serve as an efficient and lightweight fruit detector in fruit detection.

### 3.2. Comparison of Fruit Tracking Performance

**Figure 7:**
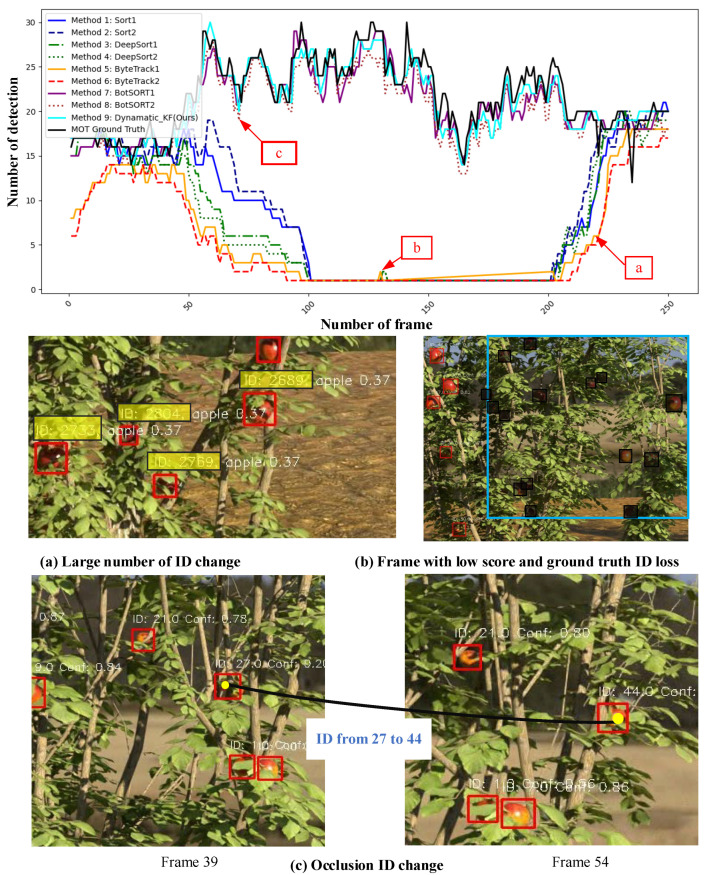
Real-time counting results by different tracking methods in video frames.

Accurate fruit counting relied on effective fruit tracking. A challenge remained with the similar appearances and significant overlap of apples in the video, leading to ID mismatch errors, as depicted in Figure 7. To enhance the tracking precision, the improved YOLOv8n detector is employed with the proposed dynamic Kalman filter method in this paper. The performance of the proposed method was compared with four common tracking algorithms: SORT, DeepSORT, ByteTrack, and BoTSORT. The comparison results between different tracking methods using multiple evaluation metrics were summarized in Table 2. The tracking and real-time counting accuracy of these methods compared to the ground true in the video frame is depicted in Figure 7, where the blue box indicated the ID loss area. With the results in Table 2 and Figure 7, the following findings can be made:

- The comparative experimental results in Table 2 demonstrated that the proposed improved YOLO detection model exhibited superior performance in recognizing individual fruits in scenes with complex backgrounds, occlusions, and overlapping fruit targets. The proposed model typically provided more accurate target positions, which helped to improve the stability and precision of subsequent tracking.
- The SORT method performed well in computation with fast processing speed and small computational cost. However, the ID switching frequency was relatively high in situations with target overlap and heavy occlusion, resulting in lower *MOTA* and *IDF1* values. By using the improved YOLO detector, the performance of SORT had improved a little with a *MOTA* of 69.7%, *IDF1* of 42.0%, and *HOTA* of 70.2%, demonstrating better overall performance.

- The robustness of the ByteTrack method in complex environments was found to be improved by further processing the high and low score detection frames. Although the overall performance of ByteTrack was slightly inferior to DeepSORT, its ability to handle low-score targets improved the continuity of target tracking. Under the Improved YOLO detector, ByteTrack achieved a *MOTA* of 75.2%, *IDF1* of 38.4, and *HOTA* of 69.1%, overcoming the typical trade-off between handling occlusions and maintaining ID consistency. This demonstrates the effectiveness of the multi-score detection approach in addressing the challenges posed by complex orchard environments, as shown in Figure 7. While the tracking methods from 1 to 6 performed relatively well in the early stage, the fluctuations in the detected and tracked fruit quantities became larger. The detection quantities were relatively low in the subsequent frames, leading to situations such as tracking loss, large ID jumps, and missed detections, shown in Figure 7a,b.
- BoTSORT was an optimization of ByteTrack that incorporated camera motion compensation, which was a crucial factor for tracking performance in dynamic environments. The experimental results showed that BoTSORT had a *MOTA* of 84.7%, *IDF1* of 55.5%, and *HOTA* of 76.4% using YOLOv8n, and a *MOTA* of 89.2%, *IDF1* of 61.9, *HOTA* of 80.2% with the Improved YOLO detector, making it a compelling choice for complex fruit tracking tasks.
- The dynamic Kalman filter tracker proposed in this study introduced a Kalman filter with a variable forget factor and combined IoU and Re-ID features to enhance target matching accuracy, effectively improving tracking accuracy and stability. The experimental results showed the proposed method outperformed other methods in indicators of *MOTA*, *IDF1*, and *HOTA*.
- As depicted in Figure 7, the overall trajectories of the curves for method 7, method 8, and the proposed method were more consistent with the ground true value, exhibiting robust detection capabilities across most frames and maintaining high tracking accuracy. However, the BoTSORT methods still exhibited undesirable changes in the identification of the same target fruit when occluded, as illustrated in Figure 7c. The Fréchet distance was employed to quantify the similarity between the curves of these methods and the ground truth curve. This metric considers both the distance between each point and the overall alignment of the curve trajectories. The results revealed that the Fréchet distance for the dynamic Kalman filter tracker proposed in this study was 5.0, significantly lower than the values of 46.5 and 24.3 for Methods 7 and 8, respectively. This demonstrates the suitability of the proposed method for the critical task of tracking and counting fruits in complex orchard environments.

### 3.3. Comparison of Fruit Counting Performance

**Figure 8:**
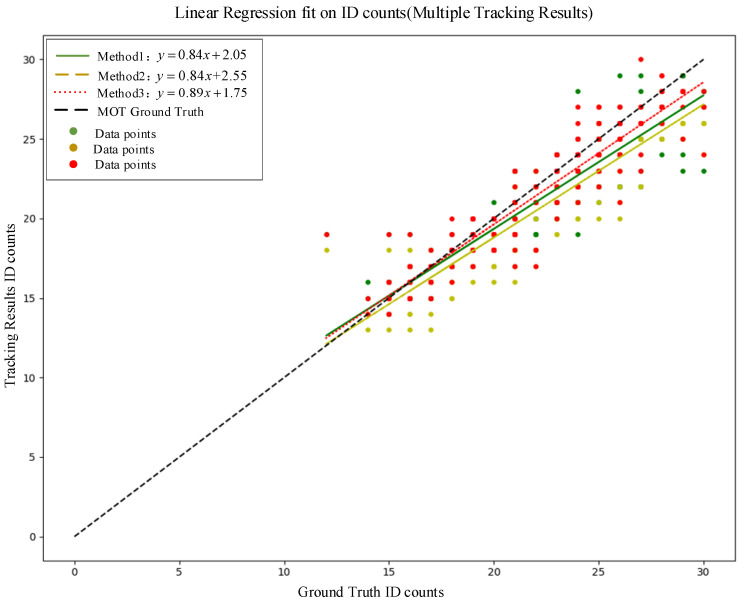
Comparison of counting regression performance.

**Figure 9:**
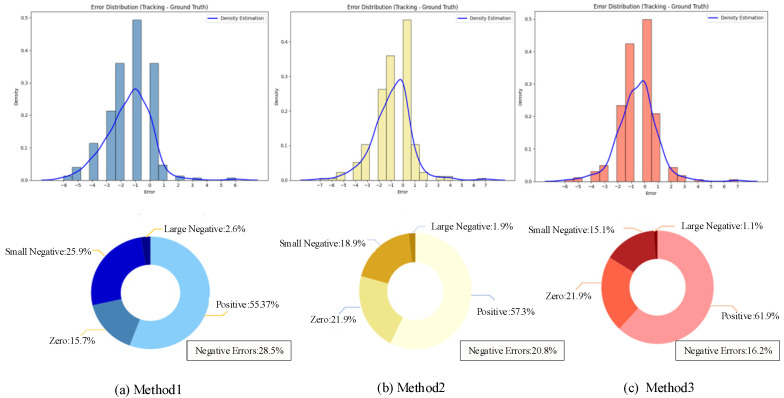
Comparison of error plot showing the distribution of ID differences.

To quantitatively evaluate the performance of different methods in fruit counting, the coefficient of determination (*R*^2^) and the root-mean-square error (RMSE) were employed to compare the counting task capabilities in Table 3. The corresponding linear regression equations were also computed. The comparison of different methods for fruit counting in regression performance and error distribution are depicted in Figure 8 and Figure 9.

In Table 3, an *R*^2^ value closer to 1 indicates a stronger goodness-of-fit between the curve and the ground truth data, while a lower RMSE signifies a smaller discrepancy between the predicted and actual values. These statistical measures provide a comprehensive assessment of the ability of these three methods to accurately track and count the fruits in the complex orchard environment.

As shown in Table 3 and Figure 7c, the BotSORT-based tracking methods (method 1 and 2) demonstrated suboptimal performance in the tracking task. They encountered issues such as occluded fruits reappearing in subsequent frames, leading to ID changes due to tracking errors. Consequently, their coefficient of determination values *R*^2^ were relatively low at 0.72 and 0.78, respectively. This demonstrates the limitations of the BoTSORT-based approaches in handling complex occlusions and maintaining consistent target identification in the challenging orchard environment. In contrast, the method proposed in this study exhibited the better performance in the target counting task. It achieved a high *R*^2^ of 0.85, indicating a robust goodness-of-fit between the predicted fruit IDs and the actual fruit counts, as depicted in Figure 8. Furthermore, its RMSE was 1.57, suggesting a small discrepancy between the estimated and actual fruit quantities, outperforming the previous two methods, as shown in Figure 9. This improvement of performance can be attributed to the target detection capabilities of the improved YOLO model and the target tracking capabilities of the dynamic Kalman filter tracker, which effectively address the challenges posed by occlusions, rapid target movements, and target interactions in the complex orchard environment. During the experiments, the target tracking of the proposed method demonstrated good stability, with minimal losses, and it was able to maintain the continuity of the targets effectively.

To further evaluate the performance of the three methods in Table 3 for fruit target tracking and counting tasks, the counting ID difference distribution of each method is presented in Figure 9. The experimental results reveal that in some frames, the original YOLOv8n detection model exhibits poor detection performance when objects are occluded, leading to fruit tracking failures and significant deviations in the ID counting of the tracking method. This underscores the importance of robust object detection as a foundation for accurate tracking. In contrast, the improved YOLO detection model effectively resolves the issue of target detection failure, exhibiting more stable detection performance and significantly improved confidence. The improved YOLO detector provided a more reliable detection basis for subsequent target tracking. However, the BotSORT algorithm proved insufficient in tracking over time, resulting in a certain accumulation of errors and a deterioration of tracking performance. This highlights the need for advanced tracking algorithms that can maintain consistent target identification even in challenging conditions. Conversely, the combination of the Improved YOLO and dynamic Kalman filter tracker reduces the negative errors to 16.2%, by fully utilizing the dynamic model for target state estimation and data association. This approach can effectively handle complex situations such as target occlusion and target recovery and thus reduce the counting error. The results demonstrated the importance of integrating robust object detection and advanced tracking techniques to achieve high-accuracy fruit counting in a complex orchard environment.

## 4. Conclusions

To address the challenges of fruit tracking and counting encountered in the complex orchard environment, this paper proposes an accurate and robust multi-target fruit tracking method to reduce the target tracking errors and ID changes caused by occlusion and rapid movement in video frames, The method combines the improved YOLO object detector with the dynamic Kalman filter tracker, which enhances the overall performance and reliability of fruit detection and tracking.

By optimizing the network structure and introducing an improved Kalman filter tracking method with a variable forget factor, the proposed method has effectively achieved accurate detection, continuous tracking, and precise counting of fruits, even in the challenging orchard conditions.

The experimental results demonstrate that the improved YOLO method achieved an exceptional *mAP*@0.5 value of 89.5% on the appledatasets, demonstrating its superior performance and reliability in fruit detection tasks. Furthermore, the dynamic Kalman filter tracker exhibited good *MOTA*, *IDF1*, and *HOTA* metrics in the tracking task, verifying the robustness and accuracy of the tracking effect. In the fruit counting task, the combination of improved YOLO and dynamic Kalman filter tracker achieved the highest *R*^2^ = 0.85 and the lowest RMSE = 1.57, providing a highly efficient and accurate solution for fruit counting in the complex orchard environment.

Based on the results provided by the experiments, the improved YOLO detector and dynamic Kalman filter tracker have emerged as the reliable solution for fruit detection, tracking, and counting. This study enriches and extends key technologies for fruit detection, multi-object tracking, and counting, providing an efficient and dependable approach for intelligent monitoring and management in complex orchard environments. It holds significant implications for agricultural automation and precision orchard management. Moving forward, this research will further explore the improvement of model real-time performance and mobile deployment to provide even more efficient and practical solutions for fruit counting systems in real-world applications.

## Figures and Tables

**Table 1:** Comparative results with different YOLO models.

Models	EfficientNetV1	MSDA	FASFFHead	*P*/%	*mAP*@0.5/%	*mAP*@0.5:0.95/%	GFLOPs
YOLOv5n	-	-	-	81.0	84.1	38.0	**4.2**
YOLOv7	-	-	-	53.1	79.0	33.1	103.5
YOLOv8n *	-	-	-	84.9	85.8	42.3	8.1
YOLOv10n	-	-	-	84.8	83.1	40.4	8.2
YOLOv11	-	-	-	85.5	84.2	41.0	6.3
YOLOv12n	-	-	-	84.8	83.5	38.7	6.0
Model 1	√			84.0	87.1	44.5	5.6
Model 2		√		83.6	86.3	42.8	8.4
Model 3			√	83.2	88.7	44.9	15.4
Model 4	√	√		85.6	87.2	44.3	5.7
Model 5	√		√	85.6	89.2	46.1	12.7
Model 6		√	√	85.7	88.3	45.2	15.4
Model 7 (proposed method)	√	√	√	**86.7**	**89.5**	**47.3**	12.9

* √ indicates the implementation of the improved modules. * Baseline: Original YOLOv8n model. Model 1: YOLOv8n with EfficientNetV1 backbone. Model 2: YOLOv8n with MSDA module. Model 3: YOLOv8n with FASFF 4-head structure. Model 4: Combination of Model 1 and Model 2 (EfficientNetV1 + MSDA). Model 5: Combination of Model 1 and Model 3 (EfficientNetV1 + FASFF). Model 6: Combination of Model 2 and Model 3 (MSDA + FASFF). Model 7 (ours): Full integration of EfficientNetV1, MSDA, and FASFF modules.

**Table 2:** Comparative results with different tracking methods.

Tracker Method	YOLOv8n	Proposed Improved YOLO	*MOTA*/%	*IDF1*/%	*HOTA*/%
SORT (method 1)	√		65.0	39.0	59.1
DeepSORT (method 2)	√		76.0	49.0	73.7
ByteTrack (method 3)	√		75.0	37.0	68.6
BoTSORT (method 4)	√		84.7	55.5	76.4
SORT (method 5)		√	69.7	42.0	70.2
DeepSORT (method 6)		√	86.7	49.7	74.2
ByteTrack (method 7)		√	75.2	38.4	69.2
BoTSORT (method 8)		√	89.2	61.9	80.2
**Dynamic Kalman filter tracker (proposed method)**		√	**95.0**	**65.5**	**82.4**

**Table 3:** Comparative results of counting performance for different methods.

Method	Tracker& Detector	*R* ^2^	RMSE	Regression Equation
1	YOLOv8 + BotSORT	0.72	2.13	y=0.84x+2.05
2	Improved YOLO + BotSORT	0.78	1.87	y=0.84x+2.55
**3**	**Improved YOLO + dynamic Kalman filter tracker**	**0.85**	**1.57**	y=0.89x+1.75

## Data Availability

The proposed method with code are available in our GitHub repository: https://github.com/zhaiyaning/Fruit_Tacking.git (accessed on 29 June 2025). The repository includes the experimental images and the implementation of the proposed method. The code was developed and tested using Python 3.8, PyTorch 1.12.1 and CUDA 11.6.
